# Real-time estimation of FES-induced joint torque with evoked EMG: Application to spinal cord injured patients

**DOI:** 10.1186/s12984-016-0169-y

**Published:** 2016-06-22

**Authors:** Zhan Li, David Guiraud, David Andreu, Mourad Benoussaad, Charles Fattal, Mitsuhiro Hayashibe

**Affiliations:** INRIA, University of Montpellier, 860 rue St Priest, Montpellier Cedex 5, 34095 France; School of Automation Engineering, University of Electronic Science and Technology of China, Xiyuan Ave 2006, Chengdu, 611731 China; Ecole Nationale d’Ingénieur de Tarbes, Tarbes, France; Centre Neurologique Mutualiste Propara [now with CRF COS DIVIO (Dijon)], Montpellier, 34090 France

**Keywords:** Functional electrical stimulation (FES), Spinal cord injured (SCI), Evoked electromyography (eEMG), Joint torque

## Abstract

**Background:**

Functional electrical stimulation (FES) is a neuroprosthetic technique for restoring lost motor function of spinal cord injured (SCI) patients and motor-impaired subjects by delivering short electrical pulses to their paralyzed muscles or motor nerves. FES induces action potentials respectively on muscles or nerves so that muscle activity can be characterized by the synchronous recruitment of motor units with its compound electromyography (EMG) signal is called M-wave. The recorded evoked EMG (eEMG) can be employed to predict the resultant joint torque, and modeling of FES-induced joint torque based on eEMG is an essential step to provide necessary prediction of the expected muscle response before achieving accurate joint torque control by FES.

**Methods:**

Previous works on FES-induced torque tracking issues were mainly based on offline analysis. However, toward personalized clinical rehabilitation applications, real-time FES systems are essentially required considering the subject-specific muscle responses against electrical stimulation. This paper proposes a wireless portable stimulator used for estimating/predicting joint torque based on real time processing of eEMG. Kalman filter and recurrent neural network (RNN) are embedded into the real-time FES system for identification and estimation.

**Results:**

Prediction results on 3 able-bodied subjects and 3 SCI patients demonstrate promising performances. As estimators, both Kalman filter and RNN approaches show clinically feasible results on estimation/prediction of joint torque with eEMG signals only, moreover RNN requires less computational requirement.

**Conclusion:**

The proposed real-time FES system establishes a platform for estimating and assessing the mechanical output, the electromyographic recordings and associated models. It will contribute to open a new modality for personalized portable neuroprosthetic control toward consolidated personal healthcare for motor-impaired patients.

## Background

Around 90 million people currently suffer from spinal cord injury (SCI) worldwide, and 85 thousand people each year survive the traumatic SCI and prepare to spend an average of 40 years or more in a wheelchair [1]. Various rehabilitation techniques and intelligent healthcare systems emerged in order to assist disabled people, releasing the heavy works from caregivers and reducing economical/social cost [2, 3]. Functional electrical stimulation (FES) is one of the neuroprosthetic techniques that can actively restore lost motor functions for motor-impaired subjects by delivering electrical current pulses to their paralyzed or weakened muscles. It may reduce patients’ dependence on assistive devices and caregivers [4, 5]. Under FES, motor units are excited synchronously [6] and action potentials are generated at motor neurons’ axons level or muscles’ fibers level, resultantly causing muscle contractions. The contractions transfer the generated force through the tendon and actuate related joints to achieve movement. FES has been applied to SCI patients, aiming to functionally assist their daily movements or training their muscles undergoing atrophy. For lower limb movements, FES is mainly dedicated to assist locomotion such as walking [7], standing [8], and correcting drop foot [9]. For upper limb movements, FES is used to help the patients to reach and grasp [10], hold [11], or lift target objects [12].

When utilizing FES, i.e. synchronous activation, the resultant joint torque was found to be highly correlated to evoked electromyography (eEMG), which brings possibility of modeling relationship between eEMG and joint torque [13–16]. Achieving accurate joint torque control using FES, is dependent on the modeling and estimation quality [17, 18]. Additionally, explicit involvement of eEMG can distinguish the actual moment elicited by stimulus and by external forces. As a result, the issue of eEMG-based torque modeling and estimation play a critical role for further implementation of joint moment control by FES.

Volitional EMG based joint movement estimation approaches are widely used on healthy subjects and good movement prediction results are reported [19, 20]. Different from able-bodied subjects [21], SCI patients are not able to generate volitional EMG due to damage of the descending efferent pathways at the spinal level. FES provides a way to synchronously recruit the motor units in place of the central nervous system. The recorded eEMG is then the summation of synchronous action potentials called M-wave. This M-wave can give pieces of information about muscle fatigue so that its tracking becomes possible [14, 22]. Some representative works were proposed to predict muscle torque under metabolic fatigue during long and interrupted stimulation with multiple fatigue-recovery cycles [23–25], aiming at modeling long-term fatigue effect of stimulated muscles when metabolic fatigue is prominent. Different from the aforementioned works on prediction of muscle torque with long recovery from metabolic consumption, our previous works were focusing on FES-induced torque offline predictions with eEMG on SCI patients both with surface and implanted FES using Kalman filter as torque predictor [22, 26], in order to predict FES-induced joint torque under potential early-middle stage fatigue without configuring recovery phase. Furthermore, we also compared different estimation methods to track muscle fatigue and improve its performance in a offline manner [27].

However, when transferring laboratory-environment-validated approaches into realistic clinical FES applications, real-time FES systems are required [7, 28, 29] and technical points should be considered. For instance, in real-time context, data acquisition, signal processing and estimation algorithms should be embedded and executed within time intervals defined by the close loop control, otherwise the effective real-time FES can not be achieved and the lag will degrade the control severely and even may induce instabilities. All the estimation approaches validated in offline are ultimately needed to be implemented in real-time FES systems to be assessed regarding their usability in a daily environment.

In this study, we develop a real-time system for estimating FES-induced torque based on eEMG for clinical scenarios. Efficient implementation of recursive Kalman filter and recurrent neural network (RNN) are used as the online estimators for identifying eEMG-to-torque relationship. Prediction results on three able-bodied subjects and three SCI patients are presented to show the performance of real-time eEMG based joint torque estimation system with Kalman filter and RNN. This framework would also open a new modality for personalized portable neuroprosthetic usage as it can capture the subject-specific contraction dynamics by the tracking of muscle activation to joint moment relationship.

## Methods

**Fig. 1:**
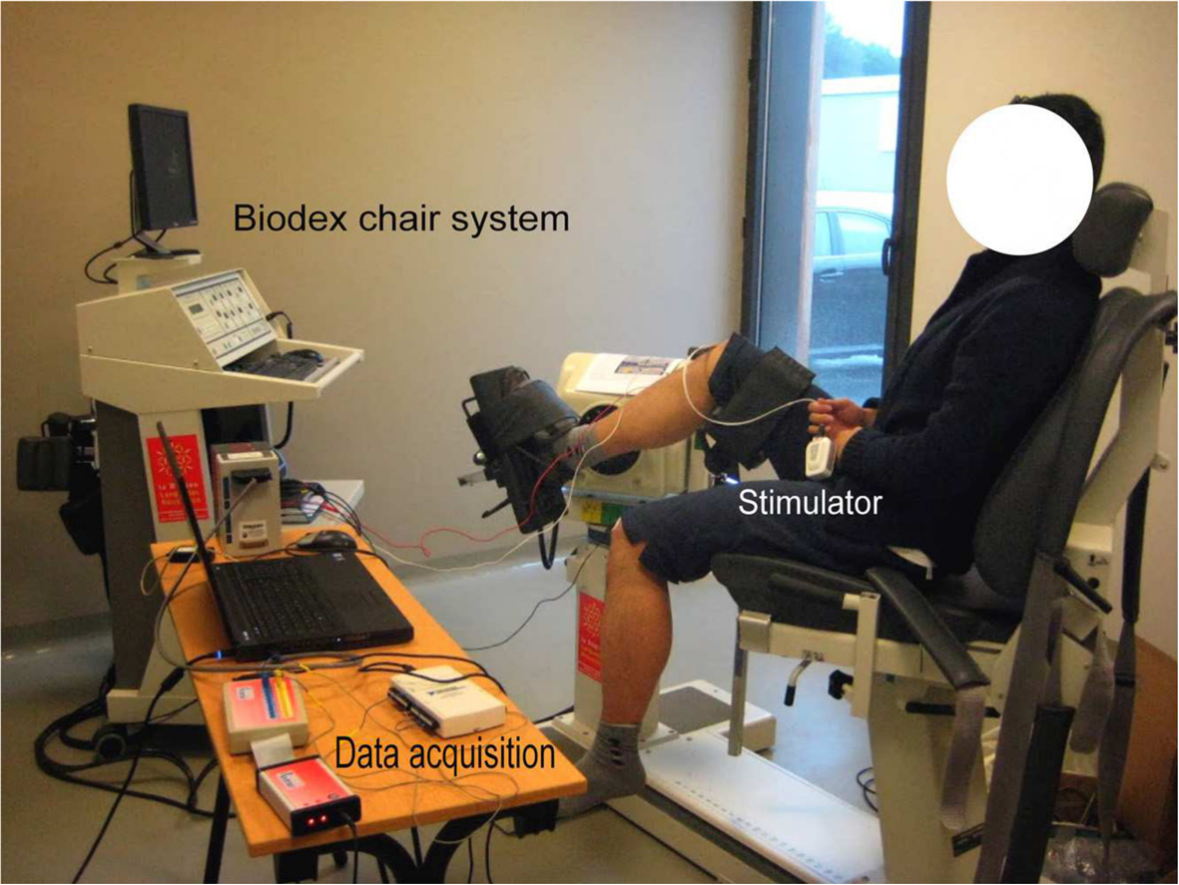
Experimental setup for real-time FES-induced torque estimation. The dynamometer is measuring the ankle torque along with EMG measurement and the stimulation is applied through wireless stimulator to TA muscle

**Fig. 2:**
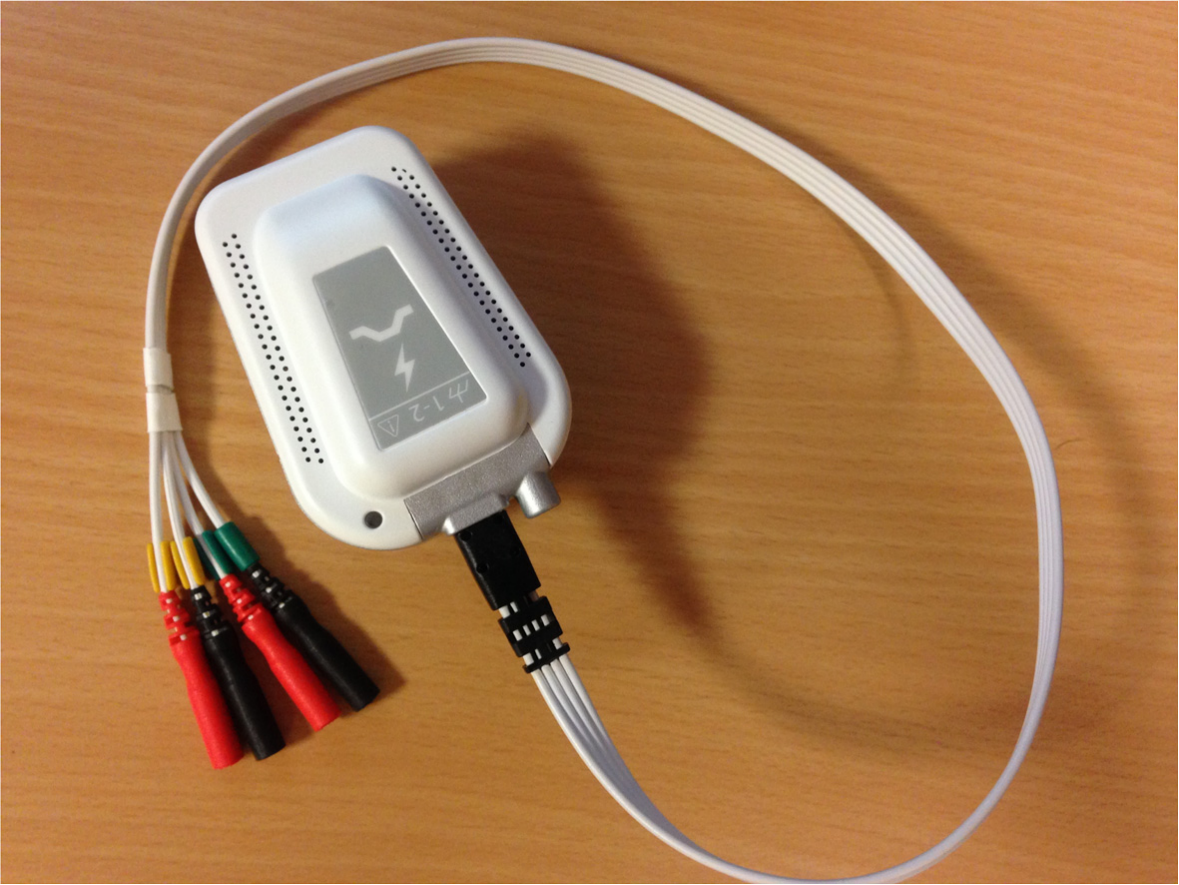
The wireless stimulator is a regulated-current dual-channel stimulator with the features as follows. Maximal electrical current is 100 mA, stimulation frequency ranges between 1 to 1000 Hz, and electrical polarity can be configured. All parameters are dynamically and remotely adjustable

**Fig. 3:**
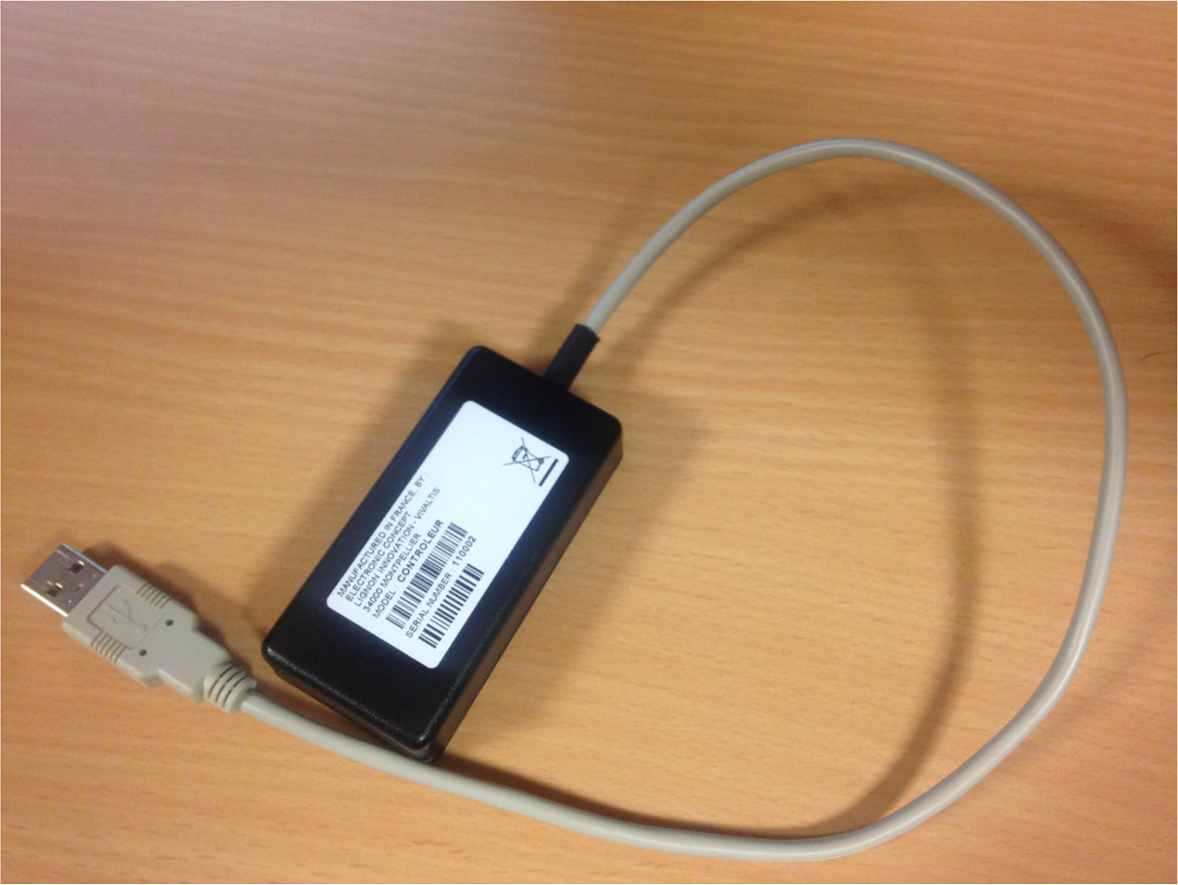
The communication/control unit of the wireless stimulator

The system tests were conducted on three able-bodied subjects and three SCI patients upon their consent with a protocol (approved by Nîmes Ethics Committee, France, 2013). In this experiment, we abide by the norms of Helsinki Declaration of 1975, revised in 2000. The SCI patients’ characteristics are shown in Table 1, and all the SCI patients have never experienced FES training until performing the experiment. Preliminary estimation results on one healthy subject through pre-testing the wireless FES system were presented in [30]. The system setup for the torque estimation experiment is shown in Fig. 1. The system consists of the wireless stimulator [31] (emanating from a transfer of our technology to Vivaltis Inc., Montpellier, France) as shown in Figs. 2 and 3, eEMG (MP100, Biopac Systems Inc., Santa Barbara, CA, USA), torque acquisition devices (Biodex 3, Shirley Corp., NY, USA), and a laptop computer with the MATLAB (version 2012a) interface for remote control of wireless stimulator and real-time data acquisition/processing. The subject/patient was seated on the chair with the ankle at 90 degrees, while the joint center was aligned with the axis of a calibrated dynamometer. The shank was adjusted with the knee joint at 40 degrees. The foot was strapped to the pedal to transmit ankle torque to the dynamometer and to allow the optimal recording of isometric ankle torque. The bipolar AgCl EMG electrodes were positioned over the muscle belly in the direction of muscle fiber with 20mm interelectrode spacing. The reference electrode was placed on the patella of contra lateral leg [27]. Electrical current pulses were delivered to the Tibialis Anterior (TA) or Medial Gastrocnemius (MG) muscle group with surface electrodes placed. Raw eEMG of TA or MG muscle group and ankle joint torque were recorded, amplified (gain 1000) and sampled at a frequency *f*_*samp*_=4096 Hz through the Biopac amplifier and an insulated 16-bit A/D national instrument card.

**Table 1:** Patient Configuration. TA represents tibialis anterior and MG represents medial gastrocnemius muscle

Patient #	Age	Weight	Height	Injury level	Time post injury	Stimulated muscle
	(year)	(kg)	(cm)	(C#/T#)	(month)	(TA/MG)
P1	24	45	172	T5	16	MG
P2	48	86	178	T10	59	MG
P3	36	64	170	C5	9	TA

The stimulation frequency *f*_*stim*_ was set between 30 and 40 Hz leading to the loop execution time between 25 ms and 33 ms. Truncation effect would appear at high stimulation frequency (up to 80 Hz) making the end of M-waves overlaps with the next forthcoming stimulation pulse. Such truncation effect becomes more obvious as fatigue level increases. The maximum pulse width (PW) of the stimulator was limited to 350 *μ*s. During all FES experiments on both healthy subjects and SCI patients, the trapezoidal stimulation train of PW was used in all sessions for all individuals for torque estimation/prediciton. In different sessions with different individuals, the plateau amplitudes of trapezoidal PW trains were randomly and specifically generated with range limited to 50 to 100 % of maximum PW. The suitable stimulation intensities were found to be from 20 to 50 mA generally and specific to each patient. The stimulation frequency for healthy subjects was chosen as 40 Hz and for SCI patients was chosen as 30 Hz, since SCI patients may be more easy to be fatigue under a higher stimulation frequency. The stimulation intensity was varying from different one to different one, and it was also subject-specific for producing stable torque. For healthy subjects, the stimulation intensity was ranging from 20 to 40 mA with their TA muscles stimulated. For patients, the stimulation intensity is ranging from 35 to 50 mA, and we have used the protocol to choose the muscle which is giving stronger response, to avoid the torque measurement problem with too small torque scale which may happen for the patients. Thus, the stimulation intensity is fixed and PW modulation is performed to have different levels of muscle contractions to induce regular joint movement. The test session included 2 phases: identification and prediction phases. Each sequence contained trapezoidal trains consisting of 2 s stimulation (0.5 s ramp-up, 1 s plateau and 0.5 s ramp-down) and 2 s rest, with 18 ±2 rest cycles. During identification phase, the plateau stimulation PW of each trapezoidal train was increased gradually with step size of from 40 to 100 % of the maximum PW. After identification, the plateau stimulation PW was randomly determined within 50 to 100 % of maximum PW in the prediction phase. The stimulation artifacts in the raw eEMG were removed by blanking with a window size at 10 ms. Further processing are: 1) Calculation of differences between adjacent raw eEMG samples recorded during one loop (the number of samplings is around *f*_*samp*_/*f*_*stim*_); 2) Comparison of these differences with a threshold, if the differences are larger than the threshold value, set the corresponding adjacent raw eEMG samples in the template window as zero. During every loop the mean absolute value (MAV) of eEMG and mean value of torque were computed based on the *f*_*samp*_/*f*_*stim*_ raw eEMG and raw torque sampling respectively, and the MAVs of eEMG were averaged to be smoother (with a about 0.8s time length sliding window). The envelope of M-wave would be specifically different from session/subject to session/subject according to stimulation intensity, pulse width, and even stimulation electrode location. Therefore we computed the mean absolute value of M-wave sampling values recorded in a fixed time window in a batch and macroscopic manner in order to make them available for input of model identification.

**Fig. 4:**
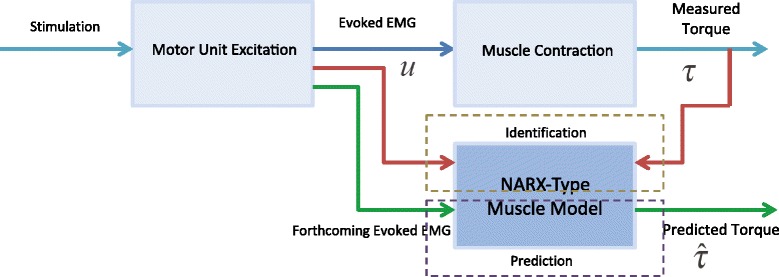
FES-induced torque identification and prediction process based on evoked EMG

Two online estimators are presented for the proposed torque estimation system. The identical models with the same orders are explored for each individual’s eEMG based torque estimation. In our previous work [27], we used these two estimators to track FES-induced torque with eEMG in offline scenario and found both of them have promising performances. In this work, we online implement them toward real-time clinical trials on SCI patients to validate and evaluate their further performances. Both estimators are computed online and have been designed to be embedded, i.e., to be later implemented in the real-time architecture of the control unit. Based on previous off-line analysis and validation [27], both of the estimators adopt NARX-type architecture to describe the muscle dynamics as shown in Fig. 4. Both estimators can guarantee their execution time within 1/*f*_*stim*_ (between 25 to 33 ms) in every stimulation loop under MATLAB2012a interface environment to enable their real-time utilization. About more details on the estimation approaches, please refer to Appendix.

## Results with discussion

**Fig. 5:**
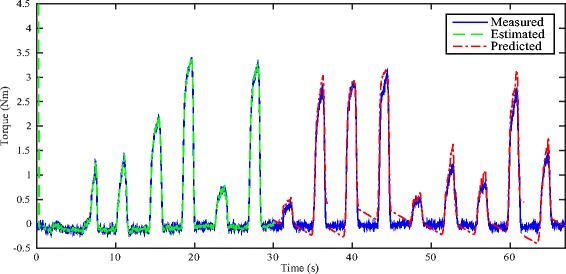
Real-time estimation/prediction results on healthy subject H1 by using NARX-RNN

**Fig. 6:**
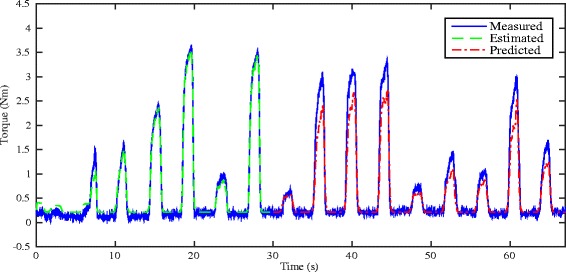
Real-time estimation/prediction results on healthy subject H1 by using Kalman filter

**Fig. 7:**
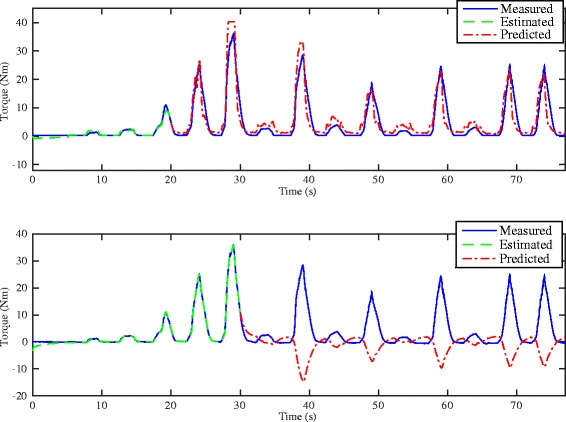
*Upper*: Real-time estimation/prediction results of the torque by Kalman filter with proper input and output amplitude normalization for the SCI patient P1. *Lower*: real-time estimation/prediction results of the torque by Kalman filter without amplitude normalization for the SCI patient P1

**Fig. 8:**
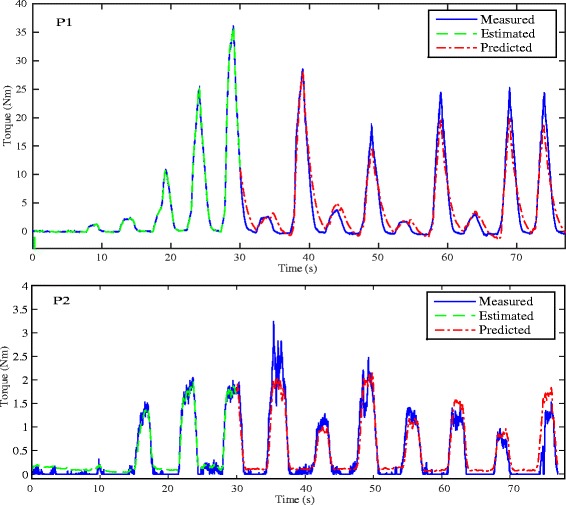
Real-time estimation/prediction results on the torque by NARX-RNN for the SCI patients P1 and P2. Patient P1 possesses strong muscle strength which was trained with daily spasticity from his neurological problem. It resulted in producing to produce the ankle joint torque in a larger range compared to patient P2, who possesses weaker muscles and thus his torque ranges at a lower level. During the experiment, we used the posture where P1 did not show the spasticity

Figures 5 and 6 respectively show the estimation/prediction results on one healthy subject H1 via NARX-RNN and Kalman filter with adequate normalization of its input and output within [0, 1]. MAV of eEMG and torque signals are normalized with their maximum measured signals respectively. From Figs. 5 and 6 we see that both methods can predict the FES-induced torque with eEMG with promising performance with VAF (variance accounted for) being 95.24 % and 93.62 % respectively. In addition to the prediction results on healthy subjects, those on SCI patients are presented: upper figure of Fig. 7 shows the real-time torque estimation results of Kalman filter with its input and output normalized within [0, 1] with respect to their maximum amplitudes respectively. The results seems promising for Kalman filter under this situation with VAF being 83.05 %. To further investigate the impact of normalization issue for Kalman filter, we used absolute scale recordings of both MAV and torque signals in the Kalman filter and NARX-RNN for comparison. Lower figure of Fig. 7 and upper figure of Fig. 8 respectively show the real-time torque estimation/prediction results by Kalman filter and NARX-RNN based on the patient P1. The input and output of the model used for identification are MAV of eEMG and torque signals without normalization. During the first 30 s, the model parameters are identified. After time instant 30 s, the torque amplitude is predicted by the previously identified model only based on MAV of eEMG. Table 2 illustrates the comparative performances of NARX-RNN and Kalman filter for predicting torque on six individuals, i.e., 3 healthy subjects (H1-H3) and 3 SCI patients (P1-P3). The input and output of the model for the Kalman filter are well normalized through trial-and-error adjusting scaling values to achieve better results, while the input and output for NARX-RNN are not normalized. The average NRMSEs of prediction by NARX-RNN and Kalman filter are respectively 10.15 % ± 6.04 % and 15.48 % ± 6.67 %, and the average VAFs of prediction by NARX-RNN and Kalman filter are respectively 85.73 % ± 7.31 % and 75.97 % ± 12.65 %. The prediction results of the 6 subjects are better with NARX-RNN than with Kalman filter. From these results, we can see that NARX-RNN may have better prediction performance than that of Kalman filter. According to aforementioned results on the 6 subjects, this implies that NARX-RNN may possess more robust estimation performance in particular when scaling of the input and output is not possible. On the contrary Kalman filter needs for pre-processing of the input and output signals (normalization within proper ranges of amplitude). When Kalman filter is to be embedded into the real-time FES system, it may limits a wide use of Kalman filter on different patients who have different strengths of muscle mechanical response under FES.

**Table 2:** Performance comparison of NARX-RNN and Kalman filter for real-time online prediction of FES-induced ankle joint torque with eEMG: root mean square errors (RMSEs), normalized root mean square errors (NRMSEs) and variance accounted for (VAF) are shown

Patient/Subject	Estimator	RMSE (Nm)	NRMSE (%)	VAF (%)
P1	NARX-RNN	2.13	6.08	92.23
	Kalman filter	6.27	17.91	83.05
P2	NARX-RNN	2.15	10.63	88.48
	Kalman filter	2.57	12.71	56.27
P3	NARX-RNN	0.24	21.24	78.75
	Kalman filter	0.31	27.31	75.16
H1	NARX-RNN	0.19	3.80	95.24
	Kalman filter	0.46	9.20	93.62
H2	NARX-RNN	1.28	10.50	77.68
	Kalman filter	1.97	15.74	69.29
H3	NARX-RNN	0.84	8.67	82.05
	Kalman filter	1.02	10.01	78.44
Average performance	NARX-RNN	1.13 ±0.87	10.15 ±6.40	85.73 ±7.31
	Kalman filter	2.10 ±2.22	15.48 ±6.67	75.97 ±12.65

Seen from Table 2, the prediction performances are more stable for healthy subjects as compared to performances of SCI patients. Although EMG signals from SCI patient are more reliable and easy to identify as no volitional contraction that may change MAV appears, muscles of SCI patients become weak to make deleted motor units inducing less progressive and stable recruitment. Moveover, the extension levels of the lesion can be drastically different from patient to patient, estimators are needed to offer more patient-specific adjustment on SCI patients than on healthy ones. It can be seen as one of the main reasons why clinical trials with SCI patients are mandatory to really validate the FES system. Again observed from Fig. 8, we could see that the NARX-RNN can deal with SCI patients with different muscle strength and clinical symptom. Patient P1 had much stronger muscle strength and put up spasticity effect in certain posture during the experiment, while patient P2 had weaker muscle strength and produced ankle joint torque of lower level.

In the current work, we have performed torque prediction tasks in isometric condition which is at neutral position of ankle angle is at 90 degree. This study is specifically focusing on the muscle response changes under FES, which can directly reflect time-variant muscle force changes. In dynamic motion situation, the torque can be altered as joint angle changes, so in non-isometric case muscle force-length and force-velocity relation should be involved. However, the potential muscle fatigue is originated from the muscle force only, thus torque prediction based on eEMG is necessary in isometric case before considering non-isometric case which needs measuring moment arm and estimating muscle force-length-velocity relation.

The concept of this work is to obtain personalized models for FES control purpose. When strong metabolic muscle fatigue arrives, FES control would not be feasible in any case in any FES system, as the muscle can not respond correctly to the stimulation. Thus, this study is targeted for early-middle fatigue stage. On FES trials among different days, even for the same subject, real muscle activation dynamics can differ a lot due to the change of stimulation contact situations. The stimulation contact situation could be drastically different due to stimulation electrode re-location. If the reality of stimulation is altered, it is necessary to make model re-identification. The proposed method can handle the different days case as well by applying this quick identification. Even with the good modeling, if the reality of stimulation is altered, we would need to make model re-identification in any case. Therefore, the quick on-line robust estimator such as RNN, could be very practical in the real world application in FES.

As compared with previous works on validation of eEMG based methods for FES-induced torque prediction from offline acquired data [22, 27], this work focuses on applying the offline-validated torque estimators into a clinical application by considering realistic implementation factors, so as to condense suitable FES approaches in specific scenarios. It will contribute to transferring offline-validated FES techniques developed in laboratory environment to clinical applications which are mostly in real-time progress. It is worth mentioning here that, concerning adaptation to wider stimulation paradigms such as stimulation intensity and stimulation frequency as new inputs of model will lead to larger model complexity and longer identification process, and eventually may degrade online performance. It is not realistic in a clinical context and not necessary to provide excessive movements. However few extensions can be considered with dual different frequencies to produce more torque in specific conditions with limited models’ complexity increase.

## Conclusion

In this paper, a real-time FES-induced torque estimation system based on a wireless portable stimulator, is proposed and developed. Kalman filter and RNN are used online to identify and then predict torque output based on eEMG recordings only. Experiment validation on 3 able-bodied subjects and 3 SCI patients show the promising performance of the proposed real-time FES system. The real-time FES-induced torque estimation system can contribute to have personalized quantitative evaluation of muscle response under FES, which is first useful for clinical diagnostics to estimate the exact mechanical response by using eEMG signal. Further, it can be useful for the implementation of real-time closed-loop FES torque control, which is previously implemented in wired stimulators and EMG system for validation [18]. Attempting to get rid of torque sensor all the time will improve portability and mobility of ambulatory autonomous closed loop FES system. The next step on which we work is to perform wireless eEMG and embedded processing on a portable controller toward personalized neuroprosthesis, and more statistic analysis is to be addressed on the real-time FES system across a diverse population of SCI patients and/or under different stimulation regimes.

## Appendix

**Kalman filter estimator**

In our previous work, a polynomial Hammerstein model (PHM) was used as the estimator model which could represent muscle contraction. We defined contraction dynamics as the relationship between muscle activation and torque production [14, 18]:

1 $$ y(k)=\sum^{l}_{i=1}a_{i}y(k-i)+\sum^{m}_{i=1}\sum^{n}_{j=0}b_{i}c_{j}u^{j}(k-i)+w(k)  $$

where *y*(*k*) denotes model response output and *u*(*k*) denotes model input, *a*_*i*_, *b*_*i*_, and *c*_*j*_ denote the model parameters to be identified, *w*(*k*) is the zero mean Gaussian white noise. In case of identifying relationship between stimulus and eEMG, *u*(*k*) denotes normalized stimulus PW and *y*(*k*) denotes MAV of eEMG; In case of identifying relationship between eEMG and torque, *u*(*k*) denotes MAV of eEMG and *y*(*k*) can denote joint torque information obtained through analog voltage signal (with the baseline-value effect eliminated).

The state space form of such PHM is described as follows:

$${ x}(k)={A}(k){x}(k-1)+{B}(k){\Phi}({u}(k-1))+{w}(k) $$

$$y(k)={x}_{1}(k) $$

where *w*(*k*) is Gaussian white noise vector and *Φ*(*u*(*k*−1))=[*u*(*k*−1) *u*^2^(*k*−1) ⋯ *u*^*n*^(*k*−1)]^*T*^. Matrix *A*(*k*) transforms the previous state vector *x*(*k*−1) into the current state vector *x*(*k*). Coefficient *B*(*k*) denotes coupled linear and nonlinear parameter matrix associated with input *u*(*k*−1).

Recursive Kalman filter with a forgetting factor *λ*∈[0.9 1] [22] is adopted as the online estimator in the real-time environment. One is estimator between stimulus and eEMG, and the other is between eEMG and torque [18]. Here, the recursive Kalman filter contains 2 parts, first one is a priori estimate phase and the second one is a posteriori update phase.

1) a priori estimate:

$$\hat{{ x}}(k)={ F}({x}(k-1),{ u}(k-1)) $$

$$\hat{{P}}(k)={A}(k-1){P}(k-1){A}^{T}(k-1)/\lambda $$

$$\hat{y}(k)=\hat{{x}}_{1}(k) $$

where $\hat {{x}}(k)$ and $\hat {{P}}(k)$ are the prior estimated state vector and plant covariance matrix respectively, and $\hat {y}(k)$ is the predicted muscle output.

2) a posteriori update:

$${S}(k)={H}(k)\hat{{P}}(k){H}^{T}(k)+\lambda {I} $$

$${K}(k)=\hat{{P}}(k){H}^{T}(k){S}^{-1}(k) $$

State vector and covariance matrix are respectively updated by

$${x}(k)\leftarrow \hat{{x}}(k)+{K}(k)({y}(k)-\hat{{y}}(k)) $$

$${P}(k)\leftarrow({I}-{K}(k){H}(k))\hat{{P}}(k) $$

Such recursive Kalman filter can be implemented with the real-time measurement and acquisition system. It is worth noting that the actual obtained torque *τ*(*k*) (Nm) is scaled according to its linear relationship to the measured analog voltage *y*(*k*) (V) exported from the analog port of the Biodex chair, where the linear scaling relationship between torque and measured analog signal is *τ*(*k*)=8.64*y*(*k*).

**Online RNN-based estimator**

The recursive NARX-RNN model which online describes muscle contraction dynamics is as follows:

2 $$ \begin{aligned} {}\tau(k)=&\sum^{l}_{r=1}v_{r}(k)\tau(k-r)+\sum^{m}_{i=1}\sum^{n}_{j=1}w_{ij}(k)u^{j}(k-i) \\&+a(k)u(k-m)\tau(k-1)+b(k)u(k-1)\tau(k-l), \end{aligned}  $$

where *k* denotes the number of stimulation loops, and torque *τ*(*k*) can be as the acquired analog torque signal without scaling conversion or actual torque amplitude.

The parameters updating at every stimulation loop is governed by:

$$\begin{array}{@{}rcl@{}} \left[ \begin{array}{c} v^{*}_{1}(k)\\ \vdots\\ v^{*}_{l}(k)\\ w^{*}_{11}(k)\\ \vdots\\ w^{*}_{mn}(k)\\ a^{*}(k)\\ b^{*}(k) \end{array}\right] =D^{\dagger}(k)\left[ \begin{array}{c} \tau(1)\\ \vdots\\ \tau(k) \end{array}\right] \end{array} $$

where *D*^*†*^(*k*) is pseudoinverse of matrix *D*(*k*), and matrix *D*(*k*)=[*Y*(*k*),*X*_1_(*k*),⋯,*X*_*m*_(*k*),*C*_1_(*k*),*C*_2_(*k*)] with

$$\begin{array}{@{}rcl@{}} Y(k)=\left[ \begin{array}{cccc} 0 & 0 & \cdots & 0 \\ \tau(0) & 0 & \cdots & 0 \\ \tau(1) & \tau(0) & \cdots & 0 \\ \vdots& \vdots & \ddots & \vdots \\ \tau(k-1) & \tau(k-2) & \cdots & \tau(k-l) \end{array}\right], \end{array} $$

$$\begin{array}{@{}rcl@{}} X_{1}(k)=\left[ \begin{array}{cccc} 0 & 0 & \cdots & 0 \\ u(0) & u^{2}(0) & \cdots & u^{n}(0) \\ \vdots& \vdots & \ddots & \vdots \\ u(k-m) & u^{2}(k-m) & \cdots & u^{n}(k-m)\\ \vdots& \vdots & \ddots & \vdots \\ u(k-1) & u^{2}(k-1) & \cdots & u^{n}(k-1) \end{array}\right], \end{array} $$

⋮

$$\begin{array}{@{}rcl@{}} X_{m}(k)= \left[ \begin{array}{cccc} 0 & 0 & \cdots & 0 \\ 0 & 0 & \cdots & 0 \\ \vdots& \vdots & \ddots & \vdots \\ 0 & 0 & \cdots & 0 \\ u(0) & u^{2}(0) & \cdots & u^{n}(0) \\ \vdots& \vdots & \ddots & \vdots \\ u(k-m) & u^{2}(k-m) & \cdots & u^{n}(k-m) \end{array}\right], \end{array} $$

$$\begin{array}{@{}rcl@{}}C_{1}(k)= \left[ \begin{array}{c} 0 \\ 0 \\ \vdots \\ u(0)\\ \vdots\\ u(k-m) \end{array}\right] \circ\left[ \begin{array}{c} 0 \\ \tau(0) \\ \vdots \\ \tau(k-1) \end{array}\right], \end{array} $$

and

$$\begin{array}{@{}rcl@{}} C_{2}(k)= \left[ \begin{array}{c} 0 \\ u(0) \\ \vdots \\ u(k-1) \end{array}\right] \circ\left[ \begin{array}{c} 0 \\ 0 \\ \vdots \\ \tau(0) \\ \vdots \\ \tau(k-l) \end{array}\right], \end{array} $$

where operator ∘ denotes Hadamard product or Schur product between vectors.

The predicted torque from *k*≥*k*_*id*_ is represented by:

$$\begin{aligned} {}\hat{\tau}(k)=&\sum^{l}_{r=1}v^{*}_{r}(k_{id})\hat{\tau}(k-r)+\sum^{m}_{i=1}\sum^{n}_{j=1}w^{*}_{ij}(k_{id})u^{j}(k-i) \\&+a^{*}(k_{id})u(k\,-\,m)\hat{\tau}(k\,-\,1)\,+\,b^{*}(k_{id})u(k\,-\,1)\hat{\tau}(k\,-\,l), \end{aligned} $$

From *k*>*k*_*id*_ the torque model is no longer updated with the last estimated parameters at *k*=*k*_*id*_.

Actually, for RNN model (2) it might not be necessary to update its parameters at each stimulation loop *k*, although it can be implemented in an online recursive way like Kalman filter. In the identification phase, which is ended at *k*=*k*_*id*_, the latest parameters are estimated for the prediction phase. In this sense, the identification could only be done once at the stimulation loop *k*=*k*_*id*_. From this point of view, rather than updating its states every loop like Kalman filter, RNN could reduce a lot of computational load. Meanwhile, RNN may need less tuning the key parameters empirically in a trial and error manner like Kalman filter, which makes it more convenient for personalized FES systems. Moreover, Kalman filter can fail to work in non-normalization scale situation, but RNN may not suffer from such problem.

## Abbreviations

FES, functional electrical stimulation; SCI, spinal cord injured (injury); eEMG, evoked electromyography; PW, pulse width; TA, Tibialis Anterior; MG, Medial Gastrocnemius; MAV, mean absolute value; RNN, recurrent neural network; PHM, polynomial Hammerstein model
